# Philopatry and Dispersal Patterns in Tiger (*Panthera tigris*)

**DOI:** 10.1371/journal.pone.0066956

**Published:** 2013-07-02

**Authors:** Digpal Singh Gour, Jyotsna Bhagavatula, Maradani Bhavanishankar, Patlolla Anuradha Reddy, Jaya A. Gupta, Mriganka Shekhar Sarkar, Shaik Mohammed Hussain, Segu Harika, Ravinder Gulia, Sisinthy Shivaji

**Affiliations:** Laboratory for the Conservation of Endangered Species, CSIR-Centre for Cellular and Molecular Biology, Hyderabad, India; University of Western Ontario, Canada

## Abstract

**Background:**

Tiger populations are dwindling rapidly making it increasingly difficult to study their dispersal and mating behaviour in the wild, more so tiger being a secretive and solitary carnivore.

**Methods:**

We used non-invasively obtained genetic data to establish the presence of 28 tigers, 22 females and 6 males, within the core area of Pench tiger reserve, Madhya Pradesh. This data was evaluated along with spatial autocorrelation and relatedness analyses to understand patterns of dispersal and philopatry in tigers within this well-managed and healthy tiger habitat in India.

**Results:**

We established male-biased dispersal and female philopatry in tigers and reiterated this finding with multiple analyses. Females show positive correlation up to 7 kms (which corresponds to an area of approximately 160 km^2^) however this correlation is significantly positive only upto 4 kms, or 50 km^2^ (r  = 0.129, p<0.0125). Males do not exhibit any significant correlation in any of the distance classes within the forest (upto 300 km^2^). We also show evidence of female dispersal upto 26 kms in this landscape.

**Conclusions:**

Animal movements are important for fitness, reproductive success, genetic diversity and gene exchange among populations. In light of the current endangered status of tigers in the world, this study will help us understand tiger behavior and movement. Our findings also have important implications for better management of habitats and interconnecting corridors to save this charismatic species.

## Introduction

Dispersal is an important event in the life history of an animal as it plays a profound role in determining the dynamics of a population, inbreeding, genetic structure, movement and continuity among subpopulations of a species. However movement of individuals (and genes) remains one of the least understood concepts in ecology and evolutionary biology [1]. There is an enormous diversity in mammalian dispersal, with significant variation between social organizations, mating systems, timing and age of dispersal, and dispersal distance. The ultimate cause of sex-biased dispersal has been explained to avoid inbreeding [2], [3], [4]. Additionally, in polygynous species, dispersal by males has also been explained by the local mate competition hypothesis as a means to reduce competition for mates [5], or to reduce competition for resources (resource competition hypothesis) among females, since females benefit from familiarity with resources in their territory and can afford better parental care [6].

Measuring sex-biased dispersal and kinship is difficult in wide-ranging, secretive mammalian species. Traditional methods rely on field observations that incorporate radiotelemetry or mark–recapture, both of which are labor intensive and usually provide data for a small sample of focal individuals [7], [8], [9]. Advances in molecular genetics now make it possible to study dispersal without extensive field data based on population level estimators [10], [11], [12]. Genetic techniques have also become effective means to determine familial relationships among individuals in a population because they employ larger sample sizes that allow for broader inferences about dispersal behaviour [11]. Measures of Fst and assignment tests are regularly used to study dispersal, immigration, emigration and structure between different populations of a species. However, genetic spatial autocorrelation tests now prove useful to detect within population dispersal and the resultant fine scale genetic structure [13]. In particular, multilocus genotype information obtained from microsatellite data, used in conjunction with spatial arrangement of individuals, has made it possible to examine the association between relatedness and dispersal [12], [14]–[17].

Dispersal in carnivores is mostly studied in social species, and data on solitary carnivores though relatively sparse show considerable inter-species as well as intra-species variations [9], [18]–[23]. Tiger (*Panthera tigris*) is endangered throughout its range and three of nine subspecies have been extirpated due to anthropogenic pressures. Despite the widespread interest in tiger, and massive efforts and investments made to recover its populations, knowledge about its behavior and ecology remains scanty. Smith, 1993 [24] observed male-biased dispersal through radio telemetry in a few random animals. Due to scarcity, extensive range, and secretiveness, dispersal was never studied extensively in any of the extant six tiger subspecies and there exists a lacuna on empirical information on tiger dispersal patterns across various landscapes. In light of research on other solitary carnivores which shows considerable variation on dispersal patterns even within a single species, it is now imperative to address the question of tiger dispersal and philopatry over important landscape complexes in order to make informed decisions before designing conservation programs.

We analyzed non-invasively obtained genetic data in combination with spatial autocorrelation tests and relatedness analyses to understand patterns of sex-specific dispersal and philopatry in tigers. The premise addressed here was that the presence of highly related individuals of a given sex within geographical proximity means restricted dispersal of that sex [25] which is indicative of philopatry in that sex. In this study we tested the following predictions –

1. Average relatedness among members of the philopatric sex should be higher than among individuals of the dispersing sex.
2. Relatedness distribution of individuals of the philopatric sex should be towards higher values compared to relatedness distribution of the dispersing sex.

## Materials and Methods

### Study Area and Sample Collection

**Figure 1:**
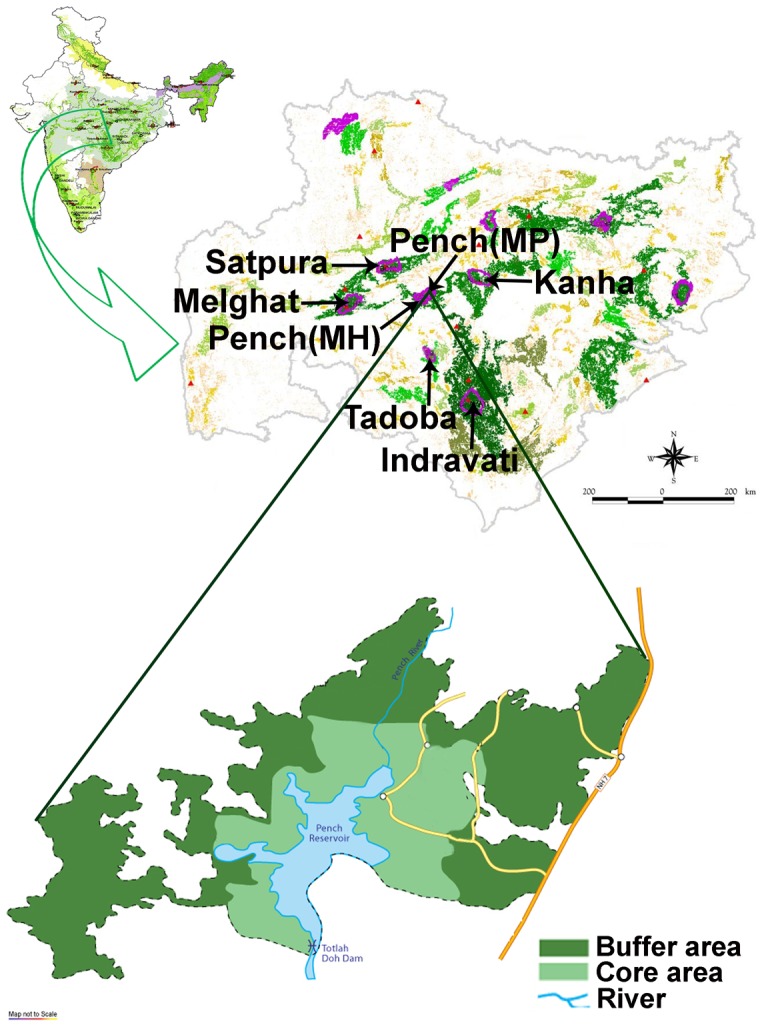
Map showing the location of Pench Tiger Reserve (PTR), Madhya Pradesh, India.

Pench Tiger Reserve (PTR) Madhya Pradesh, India is distributed between Seoni and Chhindwara districts. Core area of PTR (21°38'–21°50' North, 79°08'–79°22' East) is about 293 km^2^. PTR is one of the better managed tiger reserves in India [26] with high wild prey density (348.2/km^2^) and biomass (12384.7 kg/km^2^) [27]. The north-southwardly Pench river divides the tiger reserve into almost equal western and eastern halves (Figure 1). The forest is composed of tropical moist, dry and mixed deciduous vegetations. This reserve has a good population of tigers and co-predators like leopard (*Panthera pardus*) and wild dog (*Cuon alpinus*). Other carnivore species found in PTR are jackal (*Canis aureus*) and jungle cat (*Felis chaus*). Sloth bear (*Melursus ursinus*) is the only bear species found in this reserve [28]. Wild ungulates include chital (*Axis axis*), sambar (*Rusa unicolor*), nilgai (*Boselaphus tragocamelus*), gaur (*Bos gaurus*), barking deer (*Muntiacus muntjac*), chousingha (*Tetraceros quadricornis*) and wild pig (*Sus scrofa*) [27]–[29].

Between January and March 2010, we collected a total of 306 fresh carnivore faecal samples from the core area. During this time, 9 samples were also collected from the buffer area. All samples were collected without any preferences as it is very difficult to distinguish between adult and pre-dispersing sub-adult, or male and female faecal samples. Samples were collected with all precautions to avoid contamination, along all motorable roads and trails, in three sampling occasions with a gap of fifteen days between two sessions to allow for the deposition of fresh samples. In February 2011, 36 faecal samples were again collected from across the core area. This was done to assess and confirm long term presence of resident animals and their locations. Samples were collected in sterile, self-adhesive plastic bags (Ziploc covers) with silica beads and their geographical locations were duly recorded. On completion of field work, these samples were transported to the laboratory where they were stored at −20°C till further analysis Permission to collect tiger scat samples in PTR was granted by Principal Chief Conservator of Forests (Wildlife) and Chief Wildlife Warden, Govt. of Madhya Pradesh (letter no. 3344, dated 25^th^ June 2008).

### DNA Extraction and Individual Identification

DNA was extracted from visibly fresh faecal samples by guanidinium thiocyanate-silica method [30] with minor modifications. This was done in a dedicated room free of PCR products to minimize contamination. DNA was extracted from sets of ten samples along with an extraction control to monitor for contamination at the time of isolation. All extracts were screened by a tiger-specific PCR assay [31] and only tiger-positive samples were further analyzed. Since faecal samples yield unpredictable amounts of low quality DNA, which can lead to subsequent genotyping errors, we quantified the amount of DNA in each tiger-positive sample by real-time PCR [32]–[34]. Samples which yielded sufficient quantities of usable DNA [34] were amplified at 12 microsatellite loci (nine tetranucleotide loci - F37, F42, F53, F115, F124, F141, Fca391, Fca441, Fca424 [35]; one trinucleotide locus - E6 [31]; and two dinucleotide microsatellite loci - Fca96 [35], and E7 [31]), electrophoresed and analysed as described in Reddy *et al.* [36]. We followed the two-step multiplex PCR assay described by Arandjelovic *et al*. [37] and Reddy *et al*. [32]. All PCR steps, except addition of template DNA, were performed in a hood that was UV-irradiated before and after use to avoid contamination. All PCR reactions included positive and negative controls. PCR products from singleplex amplification step were electrophoresed on an ABI 3730 Genetic Analyser and alleles were sized relative to an internal control (500 LIZ™, Applied Biosystems) using GeneMapper software version 3.7 (Applied Biosystems). Sex of putative individuals was determined by typing zinc finger locus and randomly a few samples were rechecked with amelogenin locus [38]. Allele frequency and Identity test as implemented in CERVUS 3.0 were used to find matching pairs of genotypes. Number of loci required to distinguish individuals depends upon the variability and number of loci used. Population allelic frequencies from CERVUS 3.0 were used to determine the probability of identity (individual and sibling), or the probability that a random pair of genotypes in the population are identical to each other, but only by chance. After P_ID_ analysis, minimum number of loci required to match a pair of genotypes was kept at 8. We allowed for mismatches in up to three loci in order to rule out ‘shadow effect’ or misidentification of genotypes as a result of genotyping errors. Genotypes which mismatched at 3 or lesser number of loci were re-examined manually at the mismatching loci, in order to rule out scoring or entry errors, and in ambiguous cases, the concerned loci were genotyped again.

### Genetic Diversity Analysis

Consensus genotypes, constructed from matching pairs of genotypes, were used to determine observed and expected heterozygosities with POPGENE [39]. Observed and effective numbers of alleles [40] were also calculated using the same software. To evaluate the informativeness of heterologous loci, PIC (Polymorphic Information Content) of each locus was calculated using allelic frequencies [http://www.genomics.liv.ac.uk/animal/Pic1.html]. Tests for heterozygosity excess and deficiency were also conducted, since presence of these may confound the subsequent relatedness analyses. Deviations from Hardy-Weinberg equilibrium (HWE) were determined with GENEPOP (http://genepop.curtin.edu.au/) with default values of Markov chain parameters. Pairwise linkage disequilibrium (LD) was estimated using ARLEQUIN 3.5.1.2 [41] and allelic richness among the microsatellite loci was assessed using FSTAT 2.9.3.2 [42]. F_IS_ (inbreeding coefficient) for all animals, and males and females separately, were calculated by FSTAT.

### Spatial Autocorrelation and Relatedness Analyses

Spatial autocorrelation analysis was done with GENALEX 6.41 [43] in order to assess patterns of genetic relationships within male and female tigers. GENALEX 6.41 calculates spatial autocorrelation coefficient, r, by multivariate analysis of square of genetic distance with geographic distances. Spatial autocorrelograms were plotted with r values against distance classes. In our analysis, we plotted, autocorrelation coefficient, r, of female tigers against predefined distance classes of 3 km i.e. > = 3 km, > = 6 km upto a distance of 12 kms, and for males tigers against predefined distance classes of 4.5 km up to a distance of 13.5 kms. Since tiger territorial boundaries could not be defined with complete accuracy due to the short duration of this study, we calculated the median point of geographic coordinates of multiple genotypes of a unique individual and used this coordinate in a matrix of geographic coordinates in GENALEX 6.41. An independent study using radiotelemetry in PTR in the same time frame reported that the home range of a single adult female under observation was 44 km^2^ while that of an adult male tiger was 55 km^2^ [27]. Their study further revealed that a minimum of 25 to 30 km^2^ of undisturbed area was required for a breeding female in PTR, and we used this as a guideline in our autocorrelation analysis. Male and female genotypic and geographic data were analyzed separately. Distance classes (3 km for females and 4.5 km for males), distance interval (distance between median points of two neighbouring animals’ territories) and total distance (12 kms in females and 13.5 kms in males) were calculated according to the geometrical analysis (*πr* ^2^) of territories and total area of the tiger reserve. Test of statistical significance, p<0.05, of r values was obtained through 999 permutations and 999 bootstraps, as implemented in the software. Significance of correlograms for both male-male and female-female dyads were also checked in an advanced version of spatial autocorrelation.

Average pairwise relatedness of male-male and female-female dyads in the population were analyzed separately in order to assess patterns of dispersal and to look for evidence of kin-clustering. This was calculated with Queller and Goodnight estimator [44] as implemented in GENALEX 6.41. Values of relatedness coefficient, R, range between −1 and +1, and are indicative of the proportion of shared alleles which are identical by descent between pairs of individuals. Briefly, unrelated dyads have R value between −1 and 0.125, 2^nd^ degree relatives have values between 0.125 and 0.375, and 1^st^ degree relatives between 0.375 and 0.625. Negative R values indicate that it is highly unlikely for a pair of individuals to be related [44].

We further used ML-RELATE [45] to calculate maximum likelihood estimates of relatedness (r) [46] and relationship from codominant genetic data. This method was chosen because maximum likelihood estimates of relatedness are usually more accurate than other estimators [47] and are useful to discriminate between four common pedigree relationships: unrelated (U), half-siblings (HS), full-siblings (FS), and parent-offspring (PO).

## Results

### Individual Identification

Out of 315 faecal samples collected (306 in core area and 9 in buffer area of PTR) between January and March 2010, 113 (36%) were positively of tiger origin. DNA quantification of these samples revealed that 41 (36%) samples contained >20 pg/µl DNA, 34 (30%) samples contained 1–20 pg/µl DNA, 19 (17%) had <1 pg/µl DNA and 19 (17%) had undetectable levels of DNA [32]. Reliable genotypes were obtained for 75 (87%) of these tiger positive samples. Amplification success at twelve loci was 82.5%, average dropout rate was 4.7%, and individual multilocus genotypes were on an average 92% complete.

Twenty-eight different multilocus genotypes (individuals) were identified within the core area consisting of 22 females and 6 males. Five individuals were recaptured in the buffer zone of PTR. All individuals were captured two to eight times. Consistency of genotypes was checked with the criteria set by Arandjelovic *et al*., [37]. Twenty individuals had reliable genotypes for all 12 loci; four had genotypes for 11 loci; three for 10 loci, while only one individual had a genotype across 9 loci. Theoretical probability of two siblings sharing the same genotype, or probability of identity for siblings P_ID_(sib), was 9.02×10^−5^, and the probability of identity for two unrelated individuals P_ID_ was 2.16×10^−10^ for all the 12 loci used in this study. A high degree of discrimination power could be achieved even while considering 7 of the least variable loci with an individual probability of identity of 3.02×10^−5^ and sibling probability of identity of 5.9×10^−3^. However as the samples were not genotyped exclusively at 7 least variable loci, the chances of two different animals being misidentified as the same individual are minimal.

In 2011, a total of 36 feacal samples were randomly collected from the core area of PTR. Thirty-one (86%) of these samples were positively of tiger origin and all 31 (100%) samples yielded reliable genotypes. Sixteen unique genotypes/individuals (twelve females and four males) were identified from these samples, thirteen (eleven females and two males) of which were recaptures of animals genotyped from the previous year’s samples. We could identify one new female and two new males in the 2011 dataset. Although geographical locations were in close proximities to those of previous year’s tigers, individual genotypes identified in 2011 and their coordinates were not used in subsequent analyses in order to avoid potential bias due to sampling differences in 2010 and 2011.

### Genetic Diversity Analysis

Various measures of genetic variation are presented in Table 1. Number of alleles observed across the microsatellite loci used for all individuals varied from 3 (Fca391) to 7 (F53, Fca96, E6) with an overall mean of 5.166±1.267. Observed number of alleles (5.167) across the loci was more than effective number of alleles (3.099). Shannon’s information index and PIC showed that most of the loci were highly informative, with an overall mean polymorphism across the loci for Shannon’s information index at 1.246±0.288, and PIC at 0.593±0.124. Expected heterozygosity (He) ranged from 0.439 to 0.821 with mean of 0.655±0.120; and observed heterozygosity ranged from 0.370 to 0.926 with mean of 0.682±0.169. Average expected gene diversity [48] within the population ranged from 0.431 to 0.809 with an overall mean of 0.643±0.117 (Table 1). Loci F141 and Fca96 were not in Hardy-Weinberg equilibrium, with p<0.004 (after Bonferroni correction), which could be due to presence of relatives in the population. No two pairs of loci were found to be in linkage disequilibrium. Average allelic richness was 3.324 and average F_IS_ (inbreeding coefficient) in females was −0.05 and males −0.104 (Table 1).

**Table 1:** Measures of genetic variation at studied microsatellite loci: PTR tiger population.

Locus	Observed number of alleles	Effective number of alleles*	Shannon’s information index!	PIC#	Observed heterozygosity	Expected^a^ heterozygosity	Nei’s heterozygosity	Heterozygote deficiency^b^	Inbreeding coefficient^c^all animals	Allelic richness	Fis (Females)	Fis (Males)
F42	5	4.1303	1.501	0.7182	0.8889	0.7722	0.7579	0.1511	−0.15113	3.949	−0.171	−0.087
F115	4	2.1421	0.9887	0.4875	0.6786	0.5429	0.5332	0.2500	−0.24995	2.818	−0.245	−0.290
F141	5	2.8256	1.2026	0.5824	0.9259	0.6583	0.6461	0.4065	−0.4065	3.147	−0.588	0.111
Fca391	3	2.0918	0.8382	0.4322	0.4444	0.5318	0.5219	−0.1643	0.164347	2.365	0.167	0.211
Fca424	5	2.047	0.9374	0.4491	0.5714	0.5208	0.5115	0.0972	−0.09716	2.594	−0.119	0.063
Fca441	5	3.0825	1.2926	0.6278	0.7778	0.6883	0.6756	0.1300	−0.13003	3.428	−0.093	−0.391
E6	7	3.4922	1.4247	0.6638	0.6786	0.7266	0.7136	−0.0661	0.066061	3.602	0.057	−0.026
E7	5	1.7587	0.9021	0.4094	0.3704	0.4396	0.4314	−0.1574	0.157416	2.683	0.209	−0.143
Fca96	7	4.3653	1.6107	0.7351	0.6296	0.7855	0.7709	−0.1985	0.198472	4.132	0.231	0.13
F124	5	2.9418	1.2921	0.6123	0.7500	0.6721	0.6601	0.1159	−0.11591	3.423	−0.126	−0.081
F53	7	5.0973	1.7285	0.775	0.6250	0.8209	0.8038	−0.2386	0.238641	4.487	0.242	0.25
F37	4	3.2051	1.2364	0.6272	0.8400	0.7020	0.6880	0.1966	−0.19658	3.259	−0.174	−1.00
Mean	5.1667	3.0983	1.2463	0.5933	0.6817	0.6551	0.6428	0.0406	−0.0406	3.3239	−0.050	−0.104
St. Dev	±1.2673	±1.0339	±0.2884	±0.1236	±0.1686	±0.1203	±0.1178			±0.6501		

* Effective number of alleles [40].

! Shannon's Information index [73].

# PIC (Polymorphic Information Content).

a Expected heterozygosities were computed using Levene [74] and Nei’s [48] expected heterozygosity.

b Heterozygote deficiencies were expressed as D =  (Ho –He)/He.

c Inbreeding coefficient (Wright's) was calculated as F = 1−Ho/He.

### Spatial Autocorrelation and Relatedness Analyses

**Figure 2:**
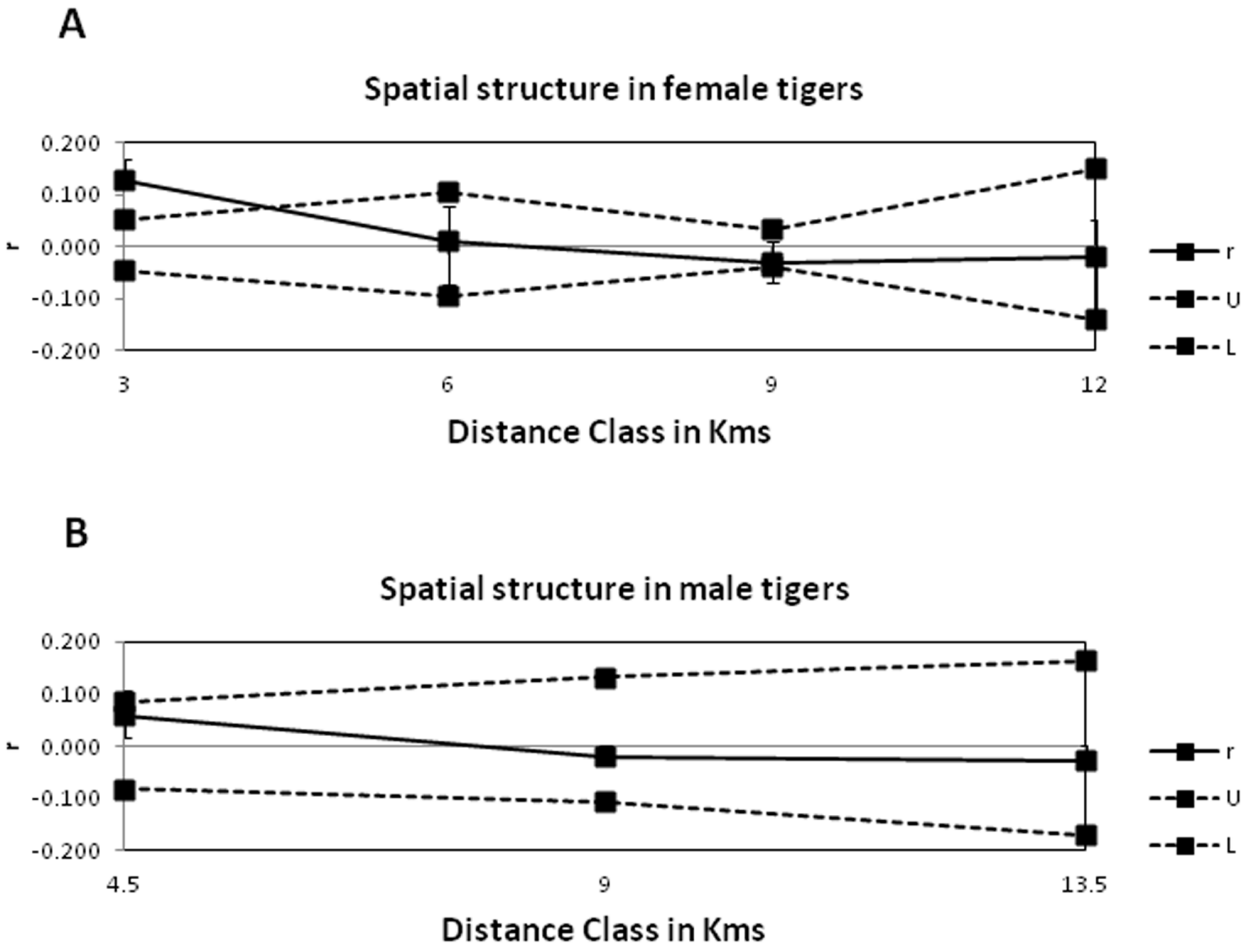
Correlogram plot of genetic correlation coefficient (*r*) as a function of distance for (A) female tigers (n  = 22) and (B) male tigers (n = 6). The permuted 999% confidence intervals (broken lines) and bootstrapped 999% confidence error bars are also shown.

Spatial autocorrelation analysis of females showed positive correlation in distance classes upto 7 kms (approximately 160 km^2^), but the autocorrelation was significantly positive only upto 4 kms (r  = 0.129 with p<0.0125 after Bonferroni correction). At distance classes greater than 3 kms but lesser than 7 kms, female autocorrelation although positive was non-significant (Figure 2A). Males did not show any significant positive autocorrelation in any of the distance classes (p>0.05) although the correlation was positive but non-significant in the distance classes up to 7 kms (Figure 2B).

Average pairwise relatedness in GENALEX for females separated from each other in the distance category 0–3 km was 0.151±0.7 (range 0.665 to 0.241). This value lies between the R values that one can expect for relatedness between 2^nd^ degree relatives. From 3 to 12 kms the average pairwise relatedness was −0.076±0.18 (range −0.425 to 0.354). On the other hand, average pairwise relatedness of male-male dyads separated by distances ranging from 0 to 13.5 kms was 0.0163±0.07. No 1^st^ degree relationships were found in male-male dyads up to 13.5 kms. Average pairwise relatedness values estimated using Queller and Goodnight mean for 231 female-female dyads was −0.044, with values ranging from 0.780 to −0.501; and for 15 male-male dyads was −0.093 with a range of 0.012 to −0.188. Maximum likelihood estimates of relatedness in ML-RELATE [45] did not show any 1^st^ degree (PO or FS) relationship among male tigers. Only two 2^nd^ degree relationships were found between six male tigers. On the other hand fourteen 1^st^ degree relationships and fourteen 2^nd^ degree relationships were found among 22 female tigers (Table 2). Fifty percent 1^st^ degree relationships and twenty-nine percent 2^nd^ degree relationships were found within 4 kms, however only fifteen percent unrelated pairs were found in the same distance.

**Table 2:** Matrix of maximum likelihood relatedness (lower triangle) and relatedness Queller and Goodnight estimator [44] (upper triangle) between PTR tigers.

	F 1	F 2	M 3	F 4	F 5	M 6	F 7	F 8	M 9	F10	F11	F12	F13	M14	F15	F16	F17	F18	F19	M20	F21	F22	F23	F24	F25	F26	F27	M28
**F 1**	0	0.14	−0.35	0.05	0.05	−0.09	0.43	0.16	0.00	0.00	−0.27	0.03	0.48	−0.03	−0.18	−0.19	−0.33	−0.37	0.09	−0.2	0.06	−0.1	0.11	−0.17	−0.31	−0.25	0.12	−0.04
**F 2**	0.03	0	−0.45	−0.03	−0.24	0.03	−0.09	−0.07	0.13	0.06	−0.31	0.00	0.06	−0.27	−0.25	−0.29	−0.11	−0.32	−0.18	−0.41	−0.07	−0.12	0.16	0.21	−0.04	−0.27	0.02	−0.19
**M 3**	0.00	0.00	0	−0.18	−0.18	−0.02	−0.25	0.15	0	−0.19	0.03	0.1	0.07	−0.06	−0.2	0.01	0	−0.07	−0.19	0	0.06	−0.03	−0.33	−0.31	−0.12	0.01	−0.14	−0.13
**F 4**	0.03	0.14	0.00	0	0.22	0.01	0.11	−0.09	0.11	0.21	−0.29	−0.09	0.15	−0.18	0.04	0	−0.03	−0.16	−0.27	0.14	−0.1	0.33	0.01	−0.13	−0.36	0	−0.23	0.32
**F 5**	0.06	0.00	0.16	0.04	0	0.09	−0.26	−0.17	−0.17	−0.11	−0.07	−0.01	0.25	−0.21	−0.03	−0.08	0.02	−0.04	0.19	0.49	0.16	0.26	0.08	−0.08	−0.36	0.13	−0.23	0.25
**M 6**	0.00	0.00	0.15	0.00	0.1	0	−0.04	−0.16	0.14	−0.1	0.24	0.01	0.1	0.07	−0.17	0.09	0.02	0.05	−0.07	0.23	0.19	0.2	0.17	0.29	0.37	−0.07	−0.24	0.06
**F 7**	0.68	0.00	0.00	0.00	0.06	0.00	0	0.07	0.5	−0.01	−0.33	−0.8	0.36	−0.07	−0.01	−0.31	−0.2	−0.08	−0.2	−0.42	0.12	0.16	0.03	0.04	0.04	−0.37	0.18	0
**F 8**	0.26	0.00	0.28	0.00	0.00	0.00	0.18	0	0.22	−0.18	−0.47	0.44	−0.03	0.06	−0.4	−0.3	−0.24	−0.27	−0.18	−0.4	0.07	−0.24	−0.1	−0.1	−0.11	−0.27	−0.13	−0.26
**M 9**	0.00	0.21	0.14	0.00	0.00	0.1	0.36	0.27	0	0.00	−0.33	−0.05	0.03	−0.27	−0.19	−0.42	0.1	0.18	−0.11	−0.14	0.17	0.53	0.1	0.28	0.13	−0.33	0.11	0.07
**F 10**	0.12	0.09	0.00	0.24	0.00	0.00	0.03	0	0.00	0	−0.52	−0.33	−0.16	−0.05	0.07	−0.24	0.13	−0.08	−0.06	0.18	−0.15	−0.03	0.46	0.13	−0.04	−0.15	−0.23	0.24
**F 11**	0.00	0.00	0.27	0.00	0.26	0.14	0.00	0.00	0.00	0.00	0	0.08	0.01	0.06	−0.08	0.15	−0.21	−0.4	−0.31	−0.22	0.06	−0.13	−0.29	−0.22	−0.35	−0.09	−0.37	−0.35
**F 12**	0.17	0.14	0.12	0.18	0.18	0.15	0.07	0.3	0.00	0.00	0.2	0	0.34	0	−0.36	−0.12	−0.27	−0.42	−0.19	−0.33	0.39	−0.07	−0.08	−0.17	−0.26	−0.04	−0.12	−0.49
**F 13**	0.44	0.25	0.15	0	0.32	0.18	0.31	0.05	0.00	0.00	0.06	0.55	0	0.19	−0.01	0.15	−0.24	−0.33	−0.44	−0.39	0.78	0.05	0.16	−0.01	−0.38	0.17	0.06	−0.11
**M14**	0.05	0.00	0.00	0.04	0.00	0.00	0.05	0.00	0.00	0.15	0.25	0.00	0.12	0	0.23	0.49	−0.39	−0.15	−0.11	−0.16	0.36	−0.22	0.04	−0.01	−0.02	0.09	−0.27	0.09
**F 15**	0.00	0.00	0.00	0.13	0.00	0.00	0.09	0.00	0.02	0.19	0.08	0.00	0.08	0.11	0	0.09	0.1	0.05	0.02	0.04	−0.31	−0.05	0.22	0.22	0.05	0.35	0.32	−0.18
**F 16**	0.00	0.00	0.15	0.18	0.03	0.01	0.00	0.00	0.00	0.13	0.26	0.04	0.00	0.44	0.07	0	−0.31	−0.05	−0.1	−0.09	0.17	−0.08	0.08	−0.18	−0.26	0.29	−0.03	−0.04
**F 17**	0.00	0.00	0.00	0.17	0.00	0.27	0.00	0.00	0.13	0.17	0.00	0.00	0.00	0.00	0.08	0.00	0	0.2	0.02	0.6	−0.13	0.34	0.01	−0.14	−0.09	−0.16	−0.04	0.06
**F 18**	0.00	0.00	0.00	0.00	0.04	0.22	0.00	0.00	0.2	0.02	0.00	0.00	0.00	0.08	0.08	0	0.33	0	−0.05	0.17	0.09	0.35	0.08	0.15	0.11	0.01	0.09	−0.06
**F 19**	0.18	0.00	0.00	0.00	0.19	0.07	0.00	0.05	0.02	0.00	0.04	0.03	0.00	0.00	0.05	0.02	0	0.00	0	0.4	−0.18	0.05	0.18	−0.05	0	−0.09	−0.02	−0.09
**M20**	0.00	0.00	0.02	0.29	0.33	0.29	0.00	0.00	0.00	0.19	0.08	0.07	0.00	0.01	0.17	0.1	0.48	0.2	0.36	0	−0.13	0.39	0.05	−0.04	−0.19	0.17	−0.37	0.24
**F 21**	0.17	0.11	0.00	0.00	0.12	0.07	0.1	0.00	0.04	0.00	0.03	0.41	0.69	0.39	0.00	0.00	0.06	0.24	0.00	0.00	0	0.14	0.15	0.03	−0.17	0.15	−0.11	−0.19
**F 22**	0.00	0.00	0.00	0.13	0.27	0.05	0.03	0.00	0.5	0.00	0.04	0.06	0.00	0.00	0.07	0.00	0.3	0.26	0.00	0.21	0.1	0	0.05	0.22	−0.05	−0.17	0.15	0.29
**F 23**	0.00	0.09	0.00	0.00	0.06	0.02	0.00	0.07	0.18	0.52	.00	0.00	0.00	0.00	0.1	0.00	0.02	0.00	0.15	0.05	0.00	0.07	0	0.49	0.09	−0.01	0	−0.09
**F 24**	0.00	0.19	0.00	0.00	0.00	0.21	0.00	0.03	0.25	0.09	0.00	0.00	0.00	0.00	0.06	0.00	0.00	0.01	0.00	0.00	0.00	0.18	0.58	0	0.66	−0.13	−0.09	−0.06
**F 25**	0.00	0.04	0.00	0.00	0.00	0.5	0.1	0.05	0.01	0.15	0.00	0.00	0.00	0.00	0.00	0.00	0.00	0.07	0.00	0.00	0.00	0.02	0.21	0.71	0	−0.32	−0.09	−0.16
**F 26**	0.00	0.00	0.03	0.00	0.16	0.13	0.00	0.00	0.00	0.00	0.04	0.2	0.07	0.08	0.3	0.27	0.04	0.08	0.01	0.24	0.11	0.00	0.00	0.00	0.00	0	0.05	−0.24
**F 27**	0.31	0.02	0.00	0.00	0.13	0.00	0.33	0.11	0.14	0.1	0.00	0.06	0.04	0.00	0.45	0.00	0.00	0.16	0.00	0.00	0.00	0.14	0.02	0.00	0.07	0.16	0	−0.33
**M28**	0.00	0.00	0.00	0.36	0.12	0.05	0.00	0.00	0.00	0.3	0.15	0.00	0.00	0.3	0.03	0.09	0.29	0.03	0.00	0.31	0.00	0.05	0.00	0.00	0.00	0.00	0.00	0

R value for unrelated dyads lies between −1 and 0.125, for 2^nd^ degree relatives between 0.125 and 0.375, and for 1^st^ degree relatives between 0.375 and 0.625.

F– female, M – male tiger.

## Discussion

Pench Tiger Reserve (PTR), Madhya Pradesh is located within one of few surviving good tiger habitats in the world and is relatively insensitive to the surrounding human matrix and human induced pressures [49]. It is also connected to other tiger breeding populations through viable corridors [50] (Figure 1). PTR therefore represents the best possible scenario for tiger persistence *in situ* [51] and also possibly is an honest reflection of uninfluenced tiger behavior in the wild. We followed a spatially focused sampling scheme which has a greater power to detect sex-biased dispersal than a spatially random method [52]. Sample collection was not biased towards any particular age or sex class. With non-invasively collected genetic data, we established the presence of 28 tigers in PTR in 2010, 22 of which were females and 6 were males. This number represents almost 100% of the existing population in PTR [50]. Our attempt to capture maximum number of animals is relevant in this study as more number of samples have a much greater positive effect on the power of spatial autocorrelation analyses to detect sex-biased dispersal than increasing the number of loci genotyped [52]. Sex ratio of 3 to 4 females: 1 male, reflects a healthy forest with substantial prey availability [28], [53]. Many of 2010 individuals were recaptured genetically in 2011 indicating a stable turnover of animals. Further, the high genetic diversity (68%) (Table 1) of PTR tiger population indicates large, long-term, stable and effective population size [54].

It is important to note in mammals, that there is some amount of dispersal even for the sex which is philopatric, although the dispersal distance for the philopatric sex is much smaller for than that of dispersing sex [55]. Mean pairwise relatedness of female tigers in close territorial proximity to each other is in the range that one expects for 1^st^ and 2^nd^ degree relatives. Therefore, although for most part, female dyads which are 1^st^ (50%) and 2^nd^ (29%) degree relatives were to be found within 4 km distance (Figure 2A), or an area of 50 km^2^, a proportion of unrelated (15%) female dyads were also found in this distance class. Further few females with 2^nd^ degree relations were found at distances upto 26 kms, thereby indicating that females also disperse. This probably reflects the connectivity and prey richness in this landscape enabling female tigers to disperse and establish new territories easily. In contrast, the near complete absence of related male dyads in this population indicates that males disperse over long distances. This corroborates with Smith’s study in 1993 in Nepal where males were seen to disperse about three times farther than females. Our spatial autocorrelation results were found to be significant in advanced autocorrelation analysis (GENALEX 6.41). Relatedness results obtained with relatedness estimators [44] were again verified by maximum likelihood estimates of relatedness [45]. This was done because maximum likelihood estimates usually have a lower root mean squared error than other estimators [47].

Thus, based on our spatial autocorrelation and relatedness analyses, although both sexes disperse, dispersal in tigers in PTR is largely male-biased, with related females in close vicinities to each other; a pattern which fits in with the resource defense hypothesis, avoidance of kin competition by males and inbreeding avoidance mechanisms which have been used to explain mammalian dispersal [4], [56]–[59]. These findings are consistent with other studies which have found dispersal in mammalian species to be primarily male-biased [5], [6]. Higher relatedness among females than among males was reported in African lion [60], [61]. Studies on solitary felids such as European lynx, cougars, bob-cats [9], [12] also suggest females may establish home ranges in their natal areas.

Dispersal studies in solitary carnivores report varying behaviors between populations and locations. Feral domestic cats show female-biased dispersal in urban areas, whereas feral domestic cats from rural areas show male dispersal [18]. Both sexes disperse equally and over equal distances in Iberian lynx [19], Canadian Lynx [21] and Eurasian lynx in Switzerland [20]. However, Eurasian lynx in Scandinavia shows male dispersal with female philopatry [23]. Genetic studies using relatedness and spatial autocorrelation analysis on some solitary carnivores show female philopatry and male dispersal, such as in bobcat [9], [12], brown bear [16], [62], racoon [63], polecat [64] and cougar [65], while genetic studies on wolverine [21] show no sex-biased dispersal.

For a solitary territorial animal like tiger, the quality of home range, like availability of water, tree cover, prey base contribute directly to fitness in females. For female tigers, males are not a limiting resource, and therefore females benefit from familiarity with territory and its resources, thereby having a direct impact on number of offspring produced, offspring survival and on quality of parental care provided. Inclusive female fitness, could in addition explain why closely related females live in close territorial proximity to each other [56], [66]. According to Sandell [57], if an area contains sufficient resources to sustain more than one female, then a system of overlapping ranges will develop especially when the population density is high. As a result, two related females (1^st^ or 2^nd^ degree relatives) might tolerate each other within their home ranges. Pressure on daughters would be lower from their mothers than from unrelated females. If a daughter can mate and have offspring, and there are enough resources in the natal area to support both, the fitness of the mother also increases [56], [66]. Male dispersal appears to have evolved as a mechanism for avoidance of kin competition and inbreeding [67]. In tigers, establishment of home ranges by males is typically required to permit breeding opportunities [68]. Theoretically, male fitness is correlated with the number of female breeding partners [58], which is usually reflected by the number of females within the male’s home range. It is likely that juvenile males disperse from their natal area because they are excluded by territorial adult males [56], [69]–[71]. Furthermore, in mammals, it has been suggested that mothers may encourage juvenile males to disperse to avoid inbreeding [56], [67].

Our data based on multilocus microsatellite analyses provide the first genetic evidence of female philopatry and male-biased dispersal in tigers. This pattern however may not be consistent in all tiger habitats. Limiting factors in this study are the number of males captured and our inability to distinguish between dispersing sub-adults and resident adults based on non-invasive DNA samples. Generally the dispersing sex incurs significant mortality costs while crossing unfavourable terrains to reach suitable habitats [72]. In mammals, survival rate can be almost 50% lower for dispersers than for philopatric individuals [72]. In light of the current endangered status of tigers in the world this study is significant in understanding tiger behavior and movement.
